# Supplementary material for: Identification of Potential Biomarkers for Diagnosis of Patients with Methamphetamine Use Disorder
Source: Int J Mol Sci. 2023 May 12;24(10):8672. doi: 10.3390/ijms24108672 (PMC10218193; doi:10.3390/ijms24108672)
Supplement: Supplementary file 1 [file ijms-24-08672-s001.zip › Supplementary table S1.pdf]

Supplementary table S1. Characteristics of all participants (n=62) included in this study.

Characteristics	Healthy controls (HC, *n*=28)	Patients with methamphetamine use disorder	
		Current patients (CP, *n*=23)	Former patients (FP, *n*=11)
Age (years, mean ± SD)	44.4 ± 7.6	46.8 ± 8.8	54.5 ± 6.0
Height (cm, mean ± SD)	173.1 ± 4.6	174.2 ± 6.2	172.5 ± 4.7
Weight (kg, mean ± SD)	74 ± 8.6	73.3 ± 12.1	76.3 ± 10.3
Body mass index (kg/m^2, mean ± SD)	24.7 ± 2.5	24.1 ± 2.8	25.6 ± 3.4
**Cigarette smoking status**			
Never-smoker (*n*, %)	9 (32)	2 (9)	0 (0)
Former smoker (*n*, %)	10 (36)	4 (17)	3 (27)
Current smoker (*n*, %)	9 (32)	17 (74)	8 (73)
**Alcohol use status**			
Non-drinker (*n*, %)	2 (7)	11 (48)	3 (27)
Moderate drinker (*n*, %)	1 (4)	0 (0)	1 (9)
Low-risk drinker (*n*, %)	3 (11)	2 (9)	1 (9)
Binge drinker (*n*, %)	22 (79)	10 (43)	6 (55)
**Drug use status**			
Methamphetamine only (*n*, %)	0 (0)	12 (52)	2 (18)
More than 2 types of drugs including methamphetamine (*n*, %)	0 (0)	11[a] (48)	9[b] (82)
**Scores of drug abuse screening tools (Korean versions)**			
**National Institute of Drug Abuse-Modified ASSIST**			
0-3 (Lower risk, *n*, %)	NA	0 (0.0)	0 (0.0)
4-26 (Moderate risk, *n*, %)	NA	6 (26.1)	1 (9.1)
27+ (High risk, *n*, %)	NA	17 (73.9)	10 (90.9)
**Drug Use Disorders Identification Test Extended**			
2~3 (Low motivate, *n*, %)	NA	2 (8.7)	1 (9.1)
4~9 (Medium motivate, *n*, %)	NA	10 (43.5)	1 (9.1)
10~14 (High motivate, *n*, %)	NA	6 (26.1)	5 (45.5)
>14 (Outlier, *n*, %)	NA	5 (21.7)	4 (36.4)
**Drug Abuse Screening Test**			
0 (Healthy, *n*, %)	NA	0 (0.0)	0 (0.0)
1-2 (Risky, *n*, %)	NA	3 (13.0)	1 (9.1)
3-5 (Harmful, *n*, %)	NA	6 (26.1)	1 (9.1)
6+ (Severe, *n*, %)	NA	14 (60.9)	9 (81.8)

[a]Cannabis (*n*=7), cocaine (*n*=2), opium (*n*=1), inhalers (e.g., toluene, butane etc., *n*=1), sedative/anesthetic drugs (e.g., diazepam, lorazepam etc., *n*=4), hallucinogens (e.g., 3,4-methylenedioxymethamphetamine, lysergic acid diethylamide etc., *n*=2), prescription stimulants (e.g., phentermine, methylphenidate etc., *n*=2), prescription opioids (e.g., morphine, codeine etc., *n*=1); [b]cannabis (*n*=8), cocaine (*n*=1), opium (*n*=1), inhalers (e.g., toluene, butane etc., *n*=2), sedative/anesthetic drugs (e.g., diazepam, lorazepam etc., *n*=2), hallucinogens (e.g., 3,4-methylenedioxymethamphetamine, lysergic acid diethylamide etc., *n*=3), γ-hydroxybutyrate (*n*=1); SD, standard deviation; NA, not analyzed.
