# Supplementary figures and images for: Identification of Potential Biomarkers for Diagnosis of Patients with Methamphetamine Use Disorder
Source: Int J Mol Sci. 2023 May 12;24(10):8672. doi: 10.3390/ijms24108672 (PMC10218193; doi:10.3390/ijms24108672)

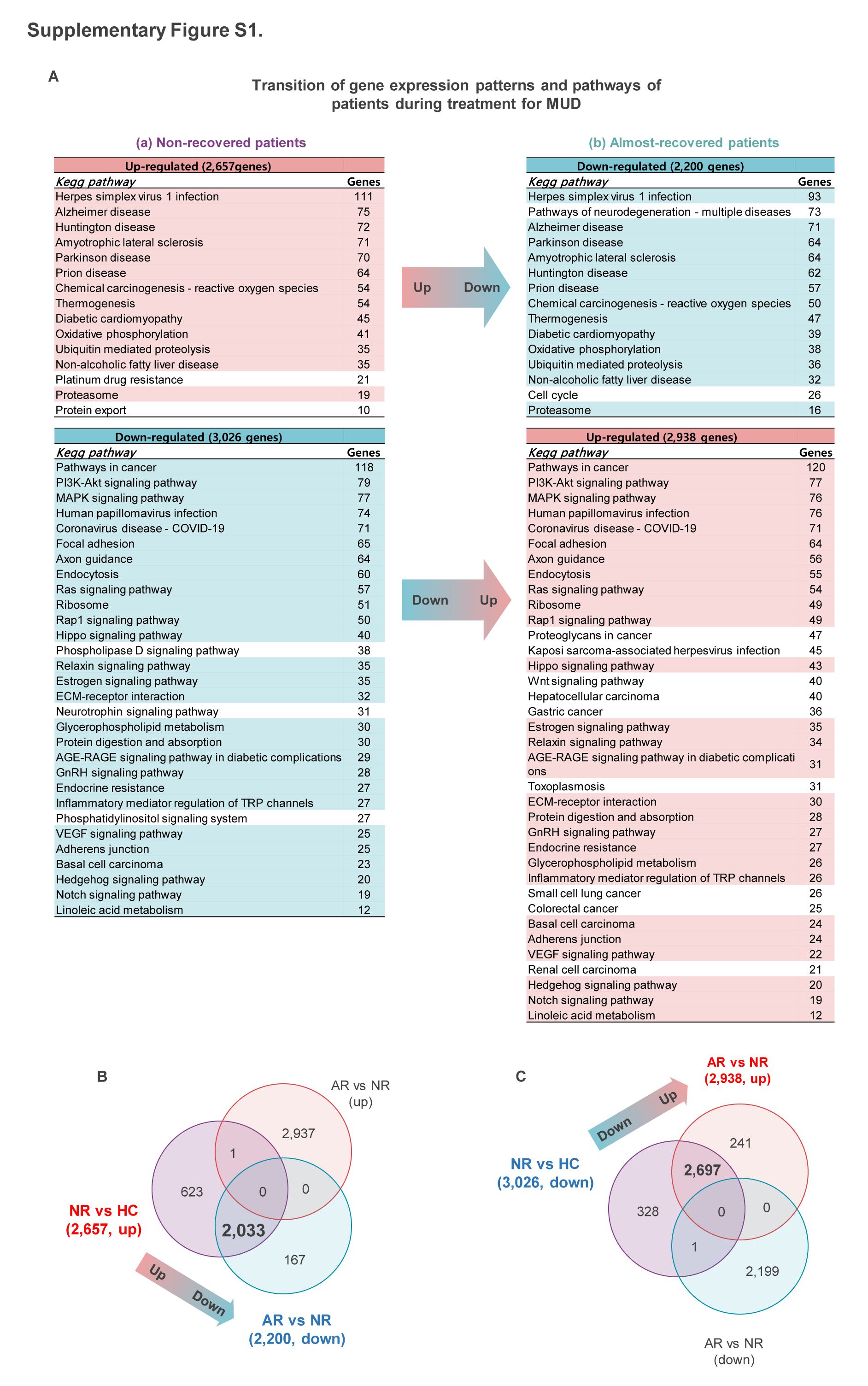

Supplement: Supplementary file 1 [file ijms-24-08672-s001.zip › Supplementary Figure S1.tif]

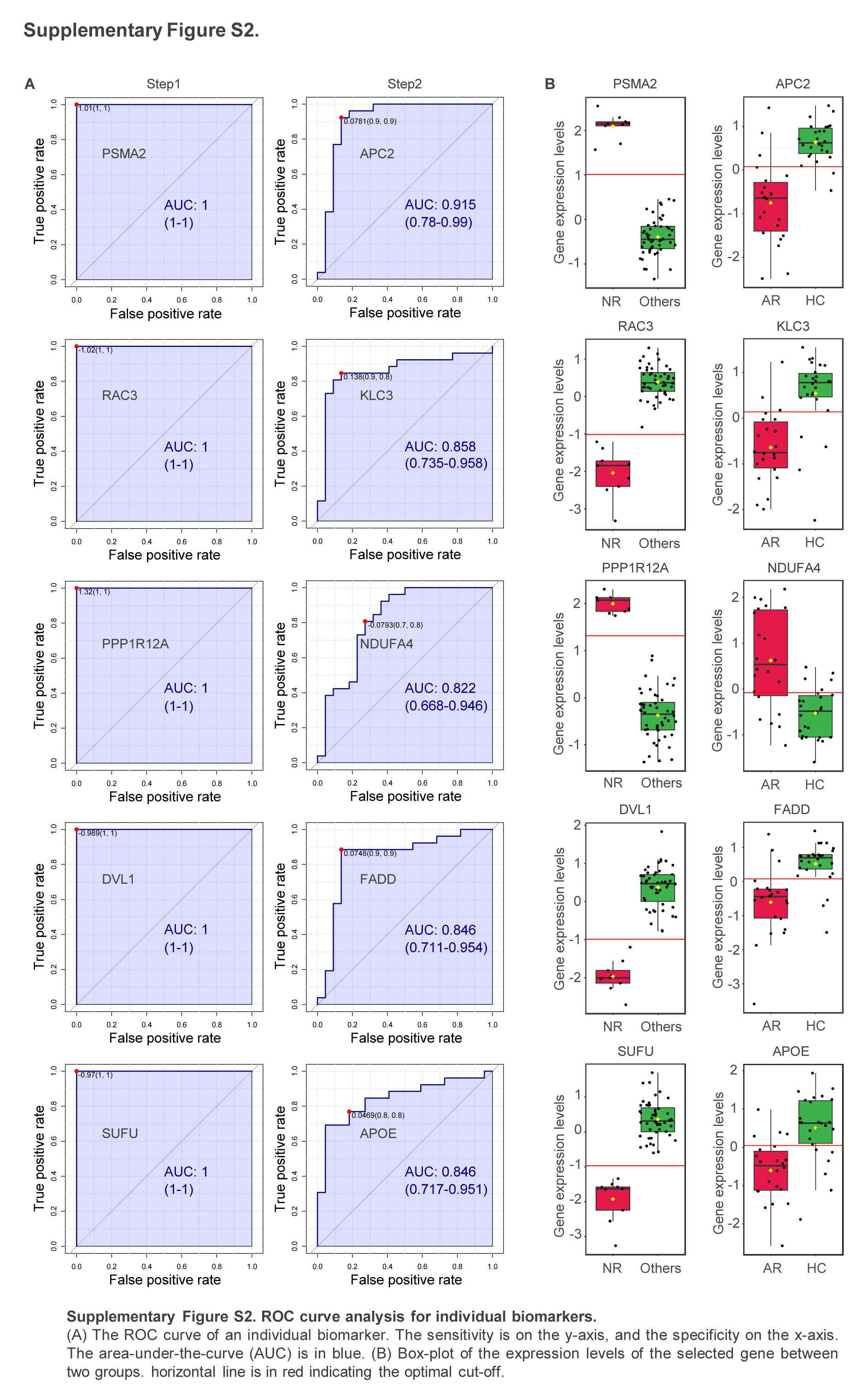

Supplement: Supplementary file 1 [file ijms-24-08672-s001.zip › Supplementary Figure S2.tif]

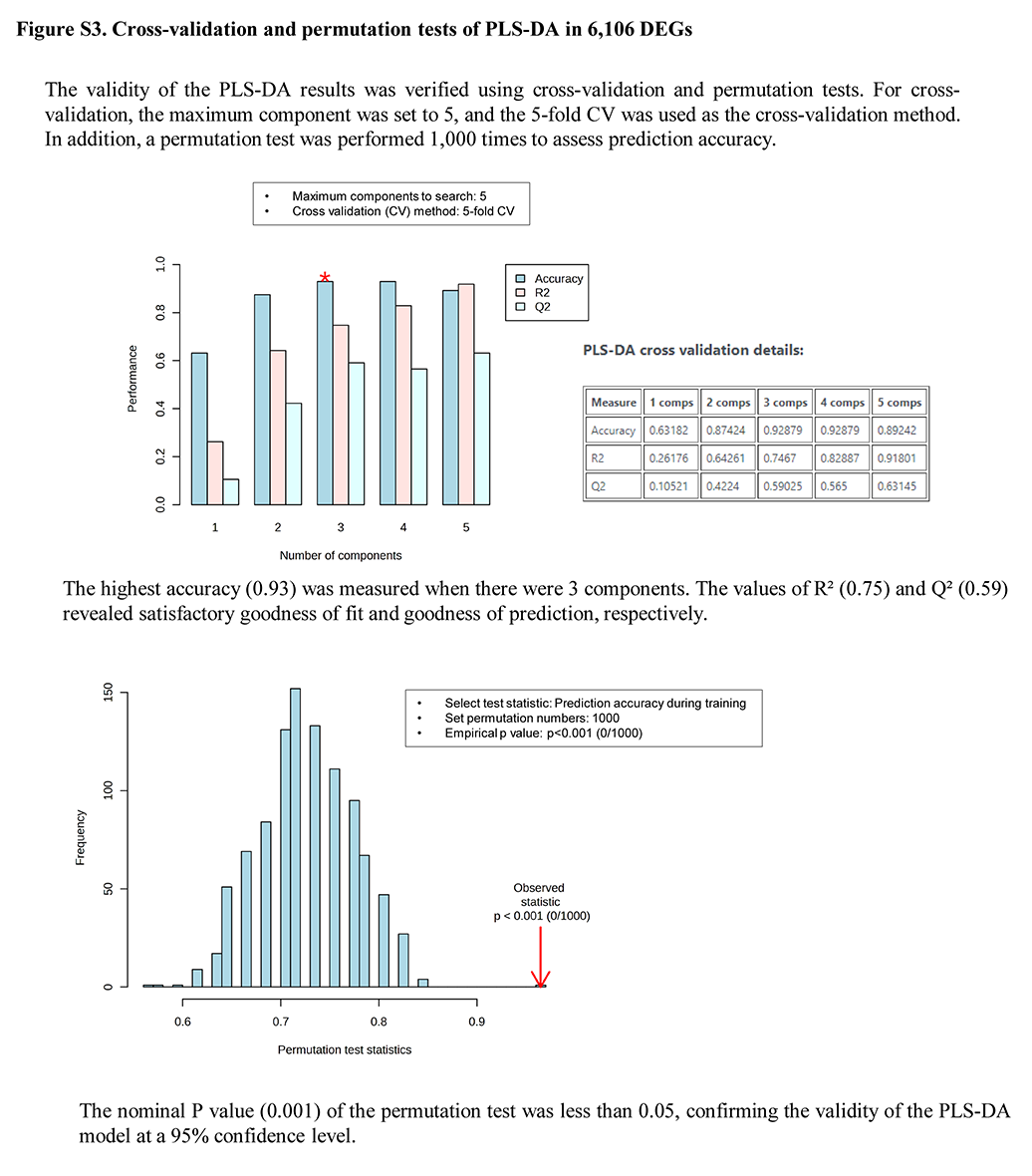

Supplement: Supplementary file 1 [file ijms-24-08672-s001.zip › Supplementary Figure S3.tif]

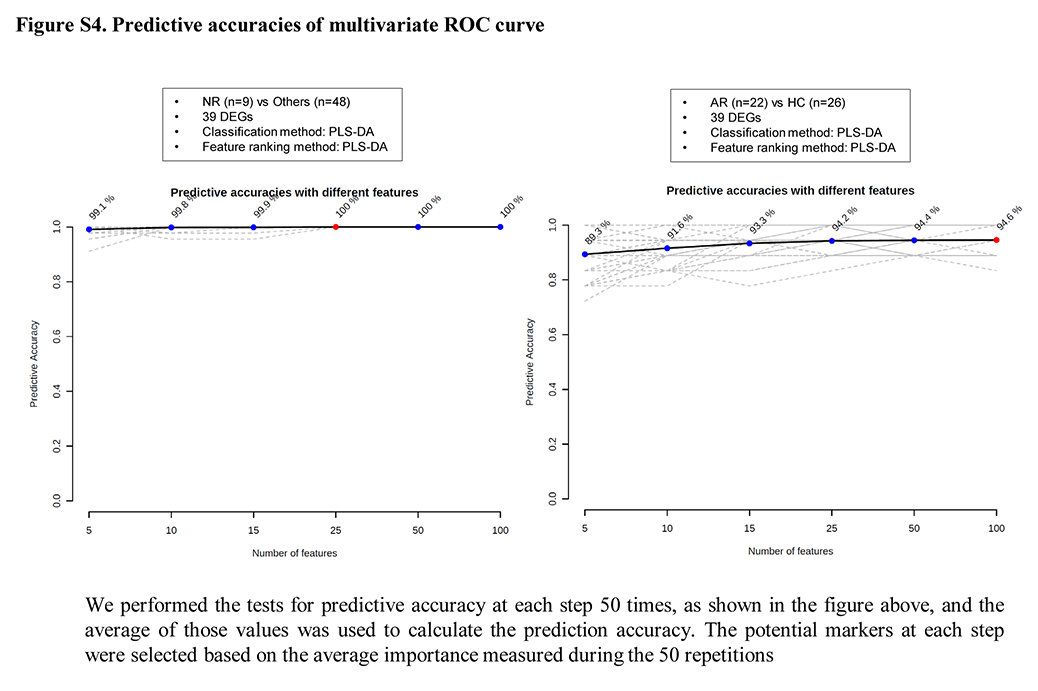

Supplement: Supplementary file 1 [file ijms-24-08672-s001.zip › Supplementary Figure S4.tif]

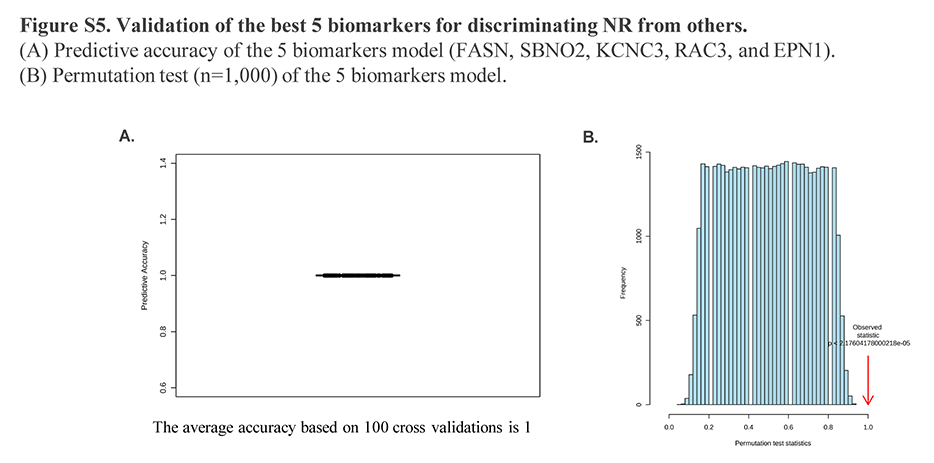

Supplement: Supplementary file 1 [file ijms-24-08672-s001.zip › Supplementary Figure S5.tif]

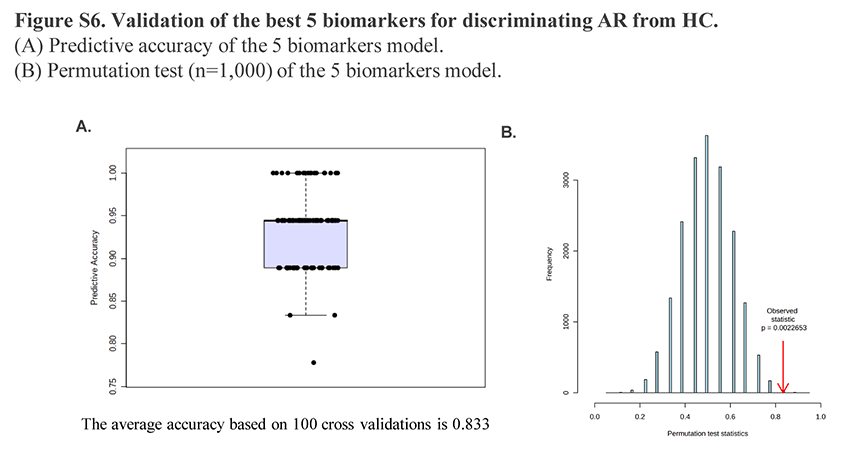

Supplement: Supplementary file 1 [file ijms-24-08672-s001.zip › Supplementary Figure S6.tif]
